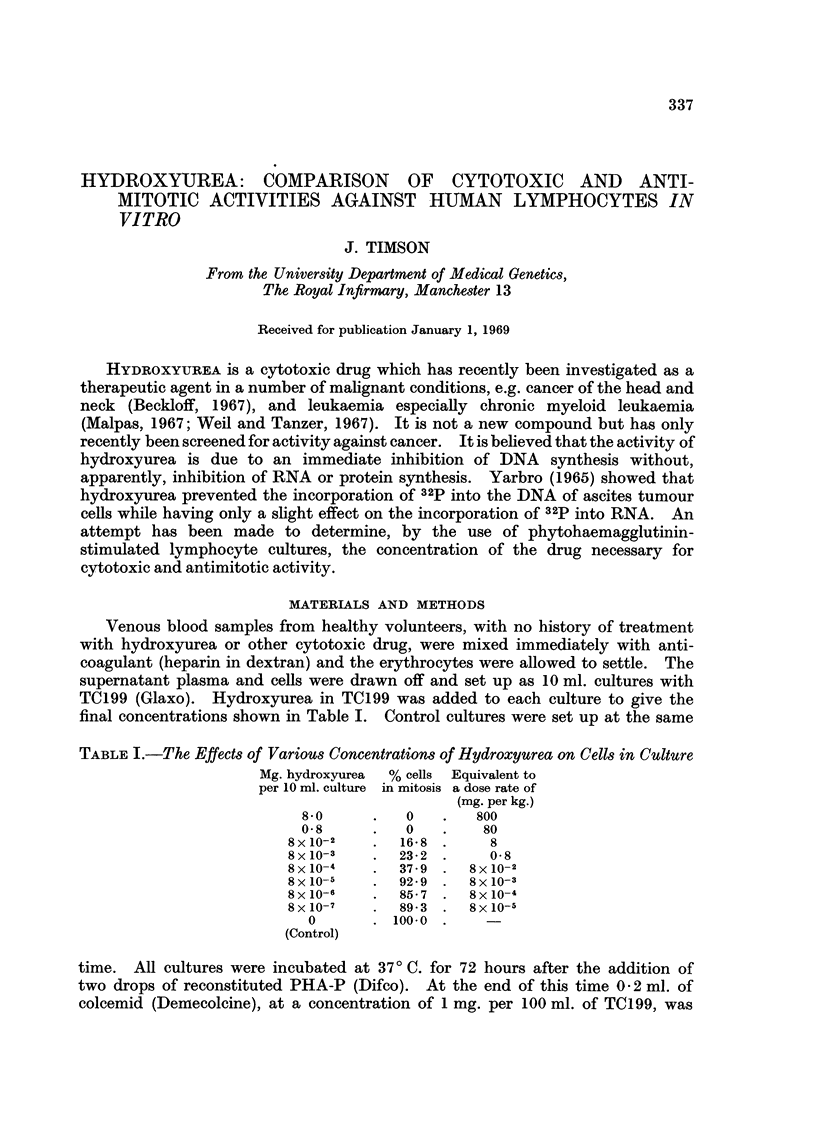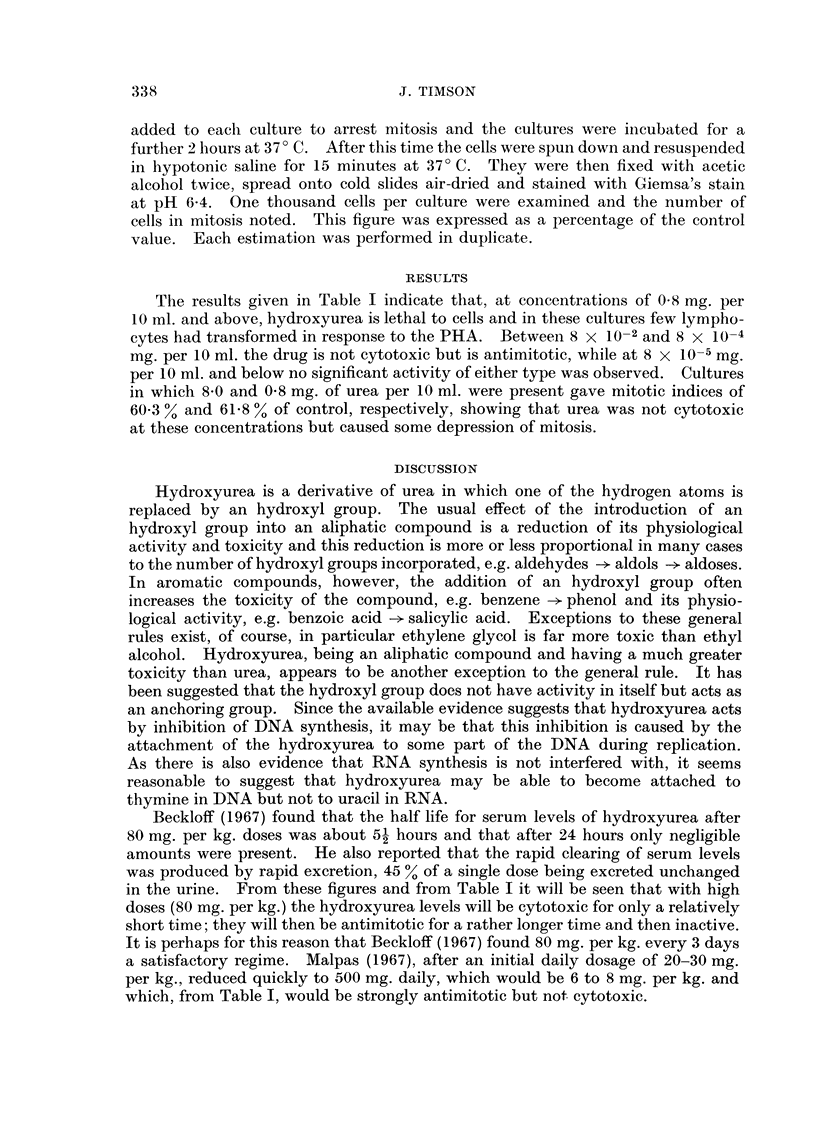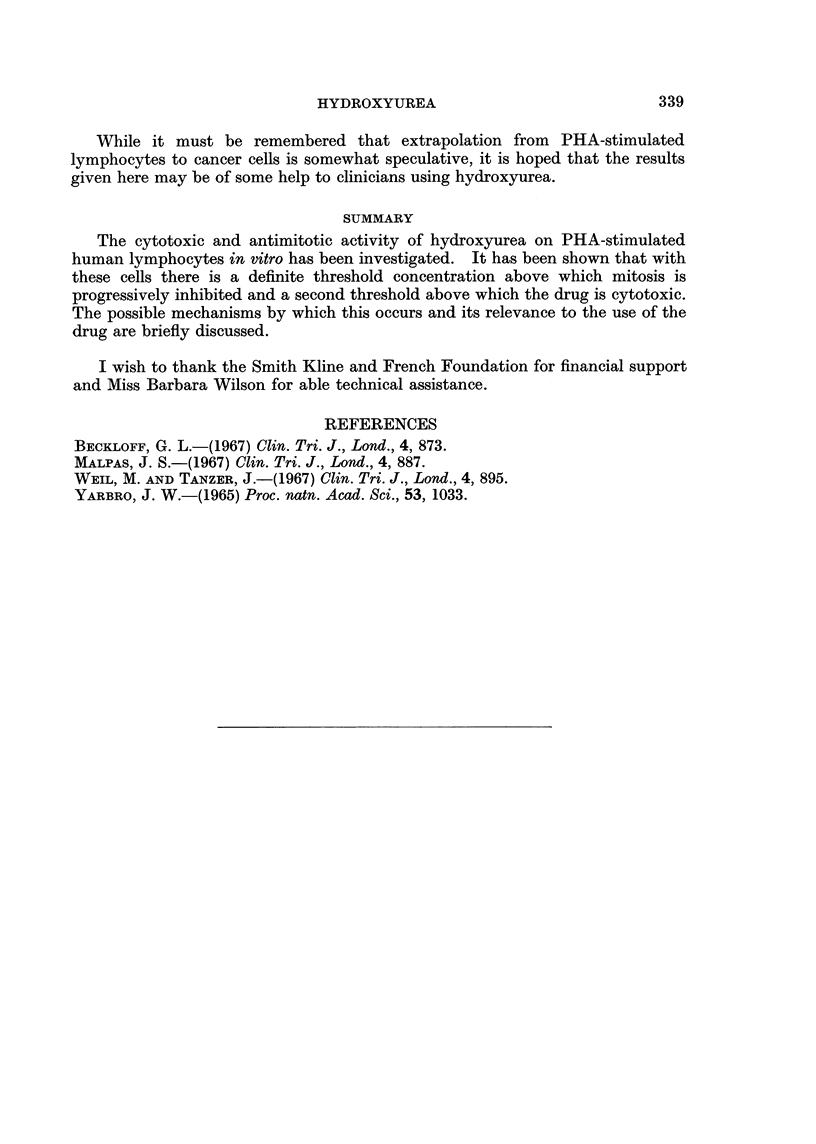# Hydroxyurea: comparison of cytotoxic and antimitotic activities against human lymphocytes in vitro.

**DOI:** 10.1038/bjc.1969.43

**Published:** 1969-06

**Authors:** J. Timson


### HYDROXYUREA: COMPARISON OF CYTOTOXIC AND ANTI-MITOTIC ACTIVITIES AGAINST HUMAN LYMPHOCYTES *IN VITRO*

J. TIMSON

*From the University Department of Medical Genetics, The Royal Infirmary, Manchester* 13

Received for publication January 1, 1969

HYDROXYUREA is a cytotoxic drug which has recently been investigated as a therapeutic agent in a number of malignant conditions, e.g. cancer of the head and neck (Beckloff, 1967), and leukaemia especially chronic myeloid leukaemia (Malpas, 1967; Weil and Tanzer, 1967). It is not a new compound but has only recently been screened for activity against cancer. It is believed that the activity of hydroxyurea is due to an immediate inhibition of DNA synthesis without, apparently, inhibition of RNA or protein synthesis. Yarbro (1965) showed that hydroxyurea prevented the incorporation of $^{32}P$ into the DNA of ascites tumour cells while having only a slight effect on the incorporation of $^{32}P$ into RNA. An attempt has been made to determine, by the use of phytohaemagglutinin-stimulated lymphocyte cultures, the concentration of the drug necessary for cytotoxic and antimitotic activity.

### MATERIALS AND METHODS

Venous blood samples from healthy volunteers, with no history of treatment with hydroxyurea or other cytotoxic drug, were mixed immediately with anti-coagulant (heparin in dextran) and the erythrocytes were allowed to settle. The supernatant plasma and cells were drawn off and set up as 10 ml. cultures with TC199 (Glaxo). Hydroxyurea in TC199 was added to each culture to give the final concentrations shown in Table I. Control cultures were set up at the same time. All cultures were incubated at 37° C. for 72 hours after the addition of two drops of reconstituted PHA-P (Difco). At the end of this time 0·2 ml. of colcemid (Demecolcine), at a concentration of 1 mg. per 100 ml. of TC199, was

TABLE I.—*The Effects of Various Concentrations of Hydroxyurea on Cells in Culture*

| Mg. hydroxyurea per 10 ml. culture | % cells in mitosis | Equivalent to a dose rate of (mg. per kg.) |
|---|---|---|
| 8·0 | 0 | 800 |
| 0·8 | 0 | 80 |
| $8\times10^{-2}$ | 16·8 | 8 |
| $8\times10^{-3}$ | 23·2 | 0·8 |
| $8\times10^{-4}$ | 37·9 | $8\times10^{-2}$ |
| $8\times10^{-5}$ | 92·9 | $8\times10^{-3}$ |
| $8\times10^{-6}$ | 85·7 | $8\times10^{-4}$ |
| $8\times10^{-7}$ | 89·3 | $8\times10^{-5}$ |
| 0 (Control) | 100·0 | — |

added to each culture to arrest mitosis and the cultures were incubated for a further 2 hours at 37° C. After this time the cells were spun down and resuspended in hypotonic saline for 15 minutes at 37° C. They were then fixed with acetic alcohol twice, spread onto cold slides air-dried and stained with Giemsa's stain at pH 6·4. One thousand cells per culture were examined and the number of cells in mitosis noted. This figure was expressed as a percentage of the control value. Each estimation was performed in duplicate.

### RESULTS

The results given in Table I indicate that, at concentrations of 0·8 mg. per 10 ml. and above, hydroxyurea is lethal to cells and in these cultures few lymphocytes had transformed in response to the PHA. Between $8 \times 10^{-2}$ and $8 \times 10^{-4}$ mg. per 10 ml. the drug is not cytotoxic but is antimitotic, while at $8 \times 10^{-5}$ mg. per 10 ml. and below no significant activity of either type was observed. Cultures in which 8·0 and 0·8 mg. of urea per 10 ml. were present gave mitotic indices of 60·3 % and 61·8 % of control, respectively, showing that urea was not cytotoxic at these concentrations but caused some depression of mitosis.

### DISCUSSION

Hydroxyurea is a derivative of urea in which one of the hydrogen atoms is replaced by an hydroxyl group. The usual effect of the introduction of an hydroxyl group into an aliphatic compound is a reduction of its physiological activity and toxicity and this reduction is more or less proportional in many cases to the number of hydroxyl groups incorporated, e.g. aldehydes → aldols → aldoses. In aromatic compounds, however, the addition of an hydroxyl group often increases the toxicity of the compound, e.g. benzene → phenol and its physiological activity, e.g. benzoic acid → salicylic acid. Exceptions to these general rules exist, of course, in particular ethylene glycol is far more toxic than ethyl alcohol. Hydroxyurea, being an aliphatic compound and having a much greater toxicity than urea, appears to be another exception to the general rule. It has been suggested that the hydroxyl group does not have activity in itself but acts as an anchoring group. Since the available evidence suggests that hydroxyurea acts by inhibition of DNA synthesis, it may be that this inhibition is caused by the attachment of the hydroxyurea to some part of the DNA during replication. As there is also evidence that RNA synthesis is not interfered with, it seems reasonable to suggest that hydroxyurea may be able to become attached to thymine in DNA but not to uracil in RNA.

Beckloff (1967) found that the half life for serum levels of hydroxyurea after 80 mg. per kg. doses was about 5½ hours and that after 24 hours only negligible amounts were present. He also reported that the rapid clearing of serum levels was produced by rapid excretion, 45 % of a single dose being excreted unchanged in the urine. From these figures and from Table I it will be seen that with high doses (80 mg. per kg.) the hydroxyurea levels will be cytotoxic for only a relatively short time; they will then be antimitotic for a rather longer time and then inactive. It is perhaps for this reason that Beckloff (1967) found 80 mg. per kg. every 3 days a satisfactory regime. Malpas (1967), after an initial daily dosage of 20–30 mg. per kg., reduced quickly to 500 mg. daily, which would be 6 to 8 mg. per kg. and which, from Table I, would be strongly antimitotic but not cytotoxic.

While it must be remembered that extrapolation from PHA-stimulated lymphocytes to cancer cells is somewhat speculative, it is hoped that the results given here may be of some help to clinicians using hydroxyurea.

### SUMMARY

The cytotoxic and antimitotic activity of hydroxyurea on PHA-stimulated human lymphocytes *in vitro* has been investigated. It has been shown that with these cells there is a definite threshold concentration above which mitosis is progressively inhibited and a second threshold above which the drug is cytotoxic. The possible mechanisms by which this occurs and its relevance to the use of the drug are briefly discussed.

I wish to thank the Smith Kline and French Foundation for financial support and Miss Barbara Wilson for able technical assistance.

### REFERENCES

BECKLOFF, G. L.—(1967) *Clin. Tri. J., Lond.*, **4**, 873.
MALPAS, J. S.—(1967) *Clin. Tri. J., Lond.*, **4**, 887.
WEIL, M. AND TANZER, J.—(1967) *Clin. Tri. J., Lond.*, **4**, 895.
YARBRO, J. W.—(1965) *Proc. natn. Acad. Sci.*, **53**, 1033.